# A Study of Deep CNN-Based Classification of Open and Closed Eyes Using a Visible Light Camera Sensor

**DOI:** 10.3390/s17071534

**Published:** 2017-06-30

**Authors:** Ki Wan Kim, Hyung Gil Hong, Gi Pyo Nam, Kang Ryoung Park

**Affiliations:** Division of Electronics and Electrical Engineering, Dongguk University, 30 Pildong-ro 1-gil, Jung-gu, Seoul 100-715, Korea; yawara18@dgu.edu (K.W.K.); hell@dongguk.edu (H.G.H.); oscar1201@dgu.edu (G.P.N.)

**Keywords:** classification of open and closed eyes, eye status tracking-based driver drowsiness detection, visible light camera, deep residual convolutional neural network

## Abstract

The necessity for the classification of open and closed eyes is increasing in various fields, including analysis of eye fatigue in 3D TVs, analysis of the psychological states of test subjects, and eye status tracking-based driver drowsiness detection. Previous studies have used various methods to distinguish between open and closed eyes, such as classifiers based on the features obtained from image binarization, edge operators, or texture analysis. However, when it comes to eye images with different lighting conditions and resolutions, it can be difficult to find an optimal threshold for image binarization or optimal filters for edge and texture extraction. In order to address this issue, we propose a method to classify open and closed eye images with different conditions, acquired by a visible light camera, using a deep residual convolutional neural network. After conducting performance analysis on both self-collected and open databases, we have determined that the classification accuracy of the proposed method is superior to that of existing methods.

## 1. Introduction

Typically, information regarding whether an eye is open or closed is used for gaze tracking systems [1], and various multimodal computer interfaces. For example, in order to measure brainwaves or accurately track eye movement, we can exclude brainwave signals or eye movements when the eyes are closed [2,3]. Furthermore, based on the results of studies that state that the blinking of Parkinson’s disease patients is slower than that of healthy people, and that of Tourette syndrome patients is faster, it is possible to predict such diseases based on the speed of opening and closing of the eyes [4]. An important use-case for detecting closed eyes is in the area of eye-tracking signal processing, where blinks might also be seen as noise to the signal [5]. To overcome this problem, they proposed a blink detection algorithm which is tailored towards head-mounted eye trackers and is robust to calibration-based variations such as eye rotation and translation [5]. Additionally, the necessity for the use of this classification in various applications is increasing, e.g., eye fatigue analysis in 3D TVs, psychological state analysis of experiment subjects, and driver drowsiness detection.

Existing studies on the classification of open and closed eyes can be largely divided into non-image-based and image-based methods. Image-based methods in turn can be classified into video-based, non-training single-image-based, and training single-image-based methods. 

Some common examples of non-image-based methods are electro-oculography (EOG) [6,7], which captures the electrical signals of the muscles around the eyes and analyzes eye movement signals, and electromyography (EMG) [8,9] and electroencephalograms (EEG) [10], which also analyze electrical signals from sensors attached to the muscles around the eyes. These methods have the advantage of fast data collection, but also have the inconveniences of needing to attach sensors to the body of a user and low accuracy when noise due to the movements of the user is picked up by the sensors. Thus, their major disadvantage lies in the imposition of restrictions on the movement of users.

Image-based methods impose fewer physical restrictions on users compared to non-image-based methods. Thus, they have the advantage of being able to obtain information from images captured at long distances. Typical examples of video-based methods within the image-based methods are optical flow in a continuous video, scale invariant feature transformation (SIFT), methods using multiple different images [11], method using eyelid and facial movements [12], and a method calculating the differences in the black pixels of the eye areas in continuous frames [13]. These methods have high accuracy because they extract features from several images. However, they require lots of time because they have to extract the necessary properties from multiple images, thus requiring extensive computations.

One of the most famous examples of a single-image-based (non-training based) method is iris detection. There have been studies on methods using the difference between the maximum iris location in a vertical histogram, assuming the existence of irises in an image, and the vertical location of the iris centers [14], a method to determine when the pupils are present via sub-block-based template matching and ellipse-fitting [15], template-matching-based methods [16], and a fuzzy-system-based method using eye segmentation [17]. The main advantage of these methods is that it is possible to distinguish between open and closed eyes in a single image without an additional training process. However, their drawback is that the performance of these methods may be negatively influenced if property extraction, which is a crucial step in the algorithms, fails to obtain the necessary information for classifying open/closed eyes from a single image. This places a limitation on the optimization of image-acquisition when extracting features. In [18], Fuhl et al. considered the task of eyelid identification and aperture estimation to estimate the mental state of subject, validate or reduce the searching regions of other eye features. For that, they proposed a novel method of eyelid identification and aperture estimation by likelihood map, which were evaluated with challenging data captured from the experiments of eye tracking performed in driving scenarios in the wild. In other research [19], Fuhl et al. newly proposed a computer vision-based algorithm to eyelids identification and aperture estimation based on the approximation of bottom and upper eyelids by oriented edges. In addition, they made the collected dataset public to other researchers so as to compare the performance. In previous research [20], they reviewed six state-of-the-art methods of pupil detection, and showed that the ellipse selector (ElSe) algorithm [21] outperformed other methods of pupil detection.

As a few examples of single-image-based (training-based) methods, there are methods based on support vector machine (SVM) [22], active appearance models (AAM) [23,24], a matching method using user-dependent eye templates [25,26], a method using tensor principal component analysis (PCA) subspace analysis [27], methods using neural networks and probability density functions [28], PCA [29], and histogram of oriented gradient (HOG)-based SVM (HOG-SVM) [30,31]. In addition, Wang et al. proposed the method of blink detection based on the trained adaptive boosting (Adaboost) with contour circle (CC) [32]. They experimentally proved that the vertical coordinate of the CC can be the best feature which can classify open/closed eyes by the linear decision surface, also.

The advantage of these methods is that they require less processing time than video-based methods and are typically more accurate at open/closed eye classification than single-image-based (non-training-based) methods. However, it is difficult to find an optimal feature extraction method and feature dimensions for extracting the features used as classifier input in these methods. Additionally, in the case of the AAM method, accurate feature extraction is difficult if the background of the analyzed image is complex or the resolution of the image is low. 

In order to resolve the problems encountered by previous studies, we consider a convolutional neural network (CNN)-based method. CNN is a deep learning method that was used to develop the multi-layer perceptron (MLP) [33]. Because of its outstanding classification capability, CNN is widely used in the image-recognition field [34], and has a great advantage in that both the filters and classifiers for optimal feature extraction are automatically acquired from training data. In contrast to CNN, the optimal filters must be manually determined by intensive experiments for conventional supervised learning-based techniques such as SVM [35] and MLP. 

There are many types of CNN models, which differ in terms of the numbers and types of layers, and the initialization method of training parameters. Some examples of CNN models are AlexNet [34] and VGG-16 [36,37], which has more layers than AlexNet, and the size of convolution filter used in VGG-16 is 3 × 3 pixels. GoogLeNet [38] uses an inception layer and a deeper structure compared to other models, and ResNet [39] has residual network and learning characteristics. We propose a method for classifying open and closed eyes by using a deep residual CNN, and compare the performance of our method with various CNN models. When compared to previous works, our research is novel in the following four ways:

- This is the first research to adopt a CNN for the classification of open and closed eye images using only a general-purpose visible light camera. By adopting a deep residual CNN, we can enhance classification performance when compared to conventional CNN structures.
- Because the optimal filter coefficients and weights for the classifier are learned during the deep residual CNN learning process, the proposed method does not require optimal threshold setting and manual filter selection for feature extraction.
- Without using a separate CNN classifier for each database (DB), and by using all DBs in a variety of environments (different images capture distances, image resolutions, image blurring, and lighting changes) for CNN learning, we implement a CNN-based classifier for open and closed eyes that can withstand different types of changes in the environment.
- By comparing the performance of the proposed method with fuzzy system-based methods, HOG-SVM based methods, and various CNN models, we demonstrate its superiority to these other models. Additionally, the self-collected database and various CNN models including the proposed deep residual CNN model trained on our experimental databases have been made public so that other researchers can compare and evaluate the performance the proposed method.

In Table 1, we present the advantages and disadvantages of the proposed and existing methods. The remainder of this paper is organized as follows: Section 2 discusses the proposed CNN-based classification method for open/closed eyes in detail. Section 3 contains analysis and explanations of our experimental results. Finally, Section 4 presents the conclusions of our research. 

## 2. Proposed Method

### 2.1. An Overview of the Proposed Approach

**Figure 1:**
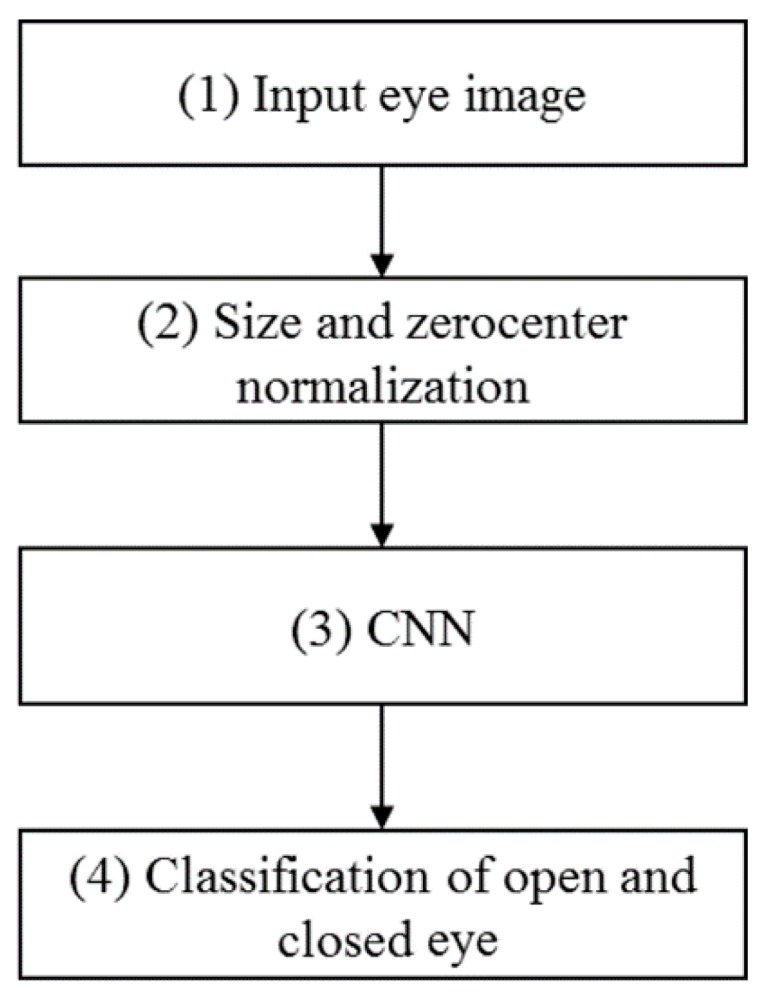
Overview of the procedure implemented by the proposed method.

Figure 1 presents a flowchart for the classification of open and closed eyes using the proposed method. First, the image of an eye is used as input, and it is resized into a standard 224 × 224 pixel image via size normalization as indicated in step (2) in Figure 1. Additionally, we perform zero-center normalization, which does not use the pixel values of the input images as they are, but instead uses the values with the mean image of the training set subtracted [40]. The normalized image is then used as the input for the pre-trained CNN and the classification of open and closed eyes is performed based on the output of the CNN. A more comprehensive explanation of the CNN operations is provided in Section 2.2.

### 2.2. The Structure of the Deep Residual CNN

We adopt the ResNet-50 CNN model [39] by revising the number of the nodes in the fully connected (*FC*) layer of the original ResNet-50 CNN model and performing fine-tuning using our experimental databases. In order to use the ResNet-50 CNN model through fine-tuning, the size of input image should be 224 × 224 pixels [39]. Therefore, we used this size of image in our research.

**Figure 2:**
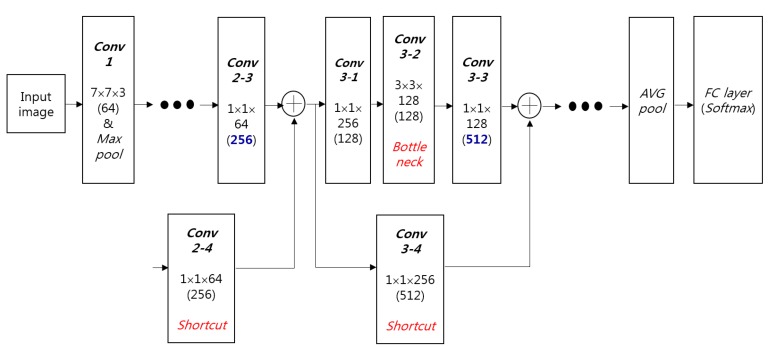
The structure of proposed CNN.

The suggested structure of the CNN is presented in Figure 2 and Table 2. Our CNN structure with regards to the number of layers, nodes, filters and filter sizes is same to that of conventional ResNet-50 CNN model [39] except for the number of output nodes in *FC layer*. Because our research aims at classifying two classes such as open and closed eyes, the number of output nodes in *FC layer* is changed into 2 from that (1000) of original ResNet-50 CNN model.

We use an eye image as the input for the CNN and perform classification of open/closed eyes based on computations within the CNN structure. The image generated after resizing and zero-center normalization is performed on the original input image is used as the final input for the CNN. In Table 2, *Conv1* ~ *Conv5* are the convolutional layers. Additionally, *Max pool* and *AVG pool* are the pooling layers that select maximum and average value respectively. They are also called subsampling layers [39,41], which operate in one of the feature abstraction stages. 

*Conv2* ~ *Conv5* are the groups of convolutional layers, and *Conv3* ~ *Conv5* include a Bottleneck structure [39]. As shown in Figure 2, the numbers of channels before and after bottleneck are 256 and 512, respectively, and they are reduced into 128 for the convolution operation by the filters of 3 × 3 × 128 for practical consideration and computational efficiency. This is the concept of bottleneck [39].

The *Conv2* ~ *Conv5* are iterated on based on the number of iterations in Table 2. Based on *Conv2* ~ *Conv5*, the convolutional layers operate sequentially. Additionally, the information in each feature map prior to *Conv2-1*, *Conv3-1*, *Conv4-1*, and *Conv5-1* is respectively element-wise summed through the shortcut layer into the output feature map of *Conv2-3*, *Conv3-3*, *Conv4-3*, and *Conv5-3* as shown in Figure 2 and Table 2. The information in the feature map prior to *Conv2-1*, *Conv3-1*, *Conv4-1*, and *Conv5-1* is called residual information. These are the main characteristics of the ResNet-50 CNN model. By also considering the residual information that is not abstracted by convolutional layers through shortcut of Figure 2, the ResNet-50 CNN model can obtain more accurate filters and classifiers. Based on these characteristics and deeper structure used in this model, the ResNet-50 CNN model shows higher accuracy of image classification compared to other famous CNN models such as VGG-16 and GoogLeNet, etc. [39], which was also confirmed by our comparative experiments in Section 3.4.

Additionally, the *FC layer* performs the same computations as those performed when applying an activation function after using the inner products in a neural network; this section is fully connected to all of the elements in its adjacent layers. The proposed method extracts the features after progressing through each of the aforementioned layers. 

These features are then passed to the *Softmax* layer [42,43]. The final classification of open and closed eye images is performed based on the output of the *Softmax* layer. The ResNet-50 CNN [39] uses batch normalization [44] after each convolutional layer. The rectified linear unit (ReLU) [42,45] is then applied as an activation function. The dropout method [34] is not used in the ResNet-50 CNN structure.

#### 2.2.1. Feature Extraction via Convolutional Layers

In the convolutional layers, feature extraction is performed by applying a traditional 2D convolution operation to the images. The range to be reflected onto the pixel values in an image varies based on the filter sizes applied. When convolution is applied, the stride values for the vertical and horizontal directions, the movement range for filter exploration based on padding options, and the width/height of resulting image change [42]. Therefore, the main factors to be considered in this layer are filter sizes, strides, padding options, and the number of filters. As shown in Table 2, *Conv1* in this method has 64 filters of size 7 × 7 × 3 and explores in the vertical and horizontal directions while striding by a two-pixel unit, and its padding is applied in three-pixel units in the vertical and horizontal directions. *Max pool* has one filter whose size is 3 × 3 pixels and explores in the vertical and horizontal directions while striding by a two-pixel unit. 

As explained in Section 2.2, *Conv2* ~ *Conv5* are the groups of convolutional layers, and *Conv3* ~ *Conv5* include a Bottleneck structure [39]. As shown in Table 2, *Conv2-1* performs the convolution operation with 64 filters of size 1 × 1 × 64, and explores in the vertical and horizontal directions while striding by a one-pixel unit. *Conv2-2* performs the convolution operation with 64 filters of size 3 × 3 × 64, and explores in the vertical and horizontal directions while striding by a one-pixel unit with padding of 1 pixel. In *Conv2* ~ *Conv5*, features are extracted in two branches as shown in Figure 2. One is the convolutional layers operating sequentially from *Conv2* ~ *Conv5*. The other is when the information in the feature map (residual information) prior to *Conv2-1*, *Conv3-1*, *Conv4-1*, and *Conv5-1* is element-wise added through the shortcut layer into the output feature map of *Conv2-3*, *Conv3-3*, *Conv4-3*, and *Conv5-3* as shown in Figure 2 and Table 2. By using small filters of size 1 × 1, the number of filter parameters requiring training is significantly reduced. 

As explained in Section 2.2, we performed the fine-tuning of the ResNet-50 CNN model [39] using our experimental databases. Fine-tuning was performed on all layers from *Conv1* to the *FC layer* in Table 2. Batch normalization is performed based on the mean and standard deviation of the data after each convolutional layer. A ReLU layer is also applied as an activation function following each batch normalization. Equation (1) is the method used to obtain the output for a given input in a ReLU layer:

(1) y=max(0,x)

where *x* and *y* are the input and output values of the ReLU function, respectively. By the Equation (1), the range of output value of *y* can be reduced as 0 or positive value, which can make the training of CNN model easier. In addition, in case that *x* is positive value, the output value of *y* is same to *x*, and the its first order derivative becomes 1, which makes the mathematical equation for training simpler. This simple equation can prevent the vanishing gradient problem [46] that can arise when a sigmoid or hyperbolic tangent function is used for learning in back-propagation [33], and has a faster computation time than a non-linear activation function. For these reasons, learning efficiency increases due to the decreased total learning time. Following *Conv5*, the *AVG pool* layer is used, where a filter of size 7 × 7 pixels is applied with a stride of 1 pixel. Thus, the size of the output feature map becomes 1 × 1 × 2048, which is used as the input to the *FC layer*.

#### 2.2.2. Classification Using One *FC Layer*

All elements in the *FC layer* are connected to all values in its adjacent layers [47]. Each connection is necessary for the computation of the weighted sum. In the *Softmax* layer, the probabilities to be used as the classification standard are calculated. Each value resulting from this layer indicates the probability of an image belonging to a certain class, and the sum of the values becomes 1. The equation used in this stage is Equation (2) [42,43]:

(2) $$\sigma {\left(z\right)}_{j}=\frac{e^{zj}}{\sum_{k=1}^{K}e^{zk}}$$

When the array of output neurons is set to *z*, the probability of the neurons belonging to the *j*th class is obtained by dividing the value of the *j*th element by the sum of the values of all the elements. Normalizing the probability to a value between 0 and 1 is considered standard for classification. By passing the input value of *z* through exponential function, the range of output value can be reduced as 0 or positive value, which can make the training of CNN model easier. In addition, by dividing the numerator with the summation of the calculated value by exponential function as shown in Equation (2), the range of output value can be normalized, which prevents the training of weights from being affected by large output value.

The final class categorization in the classification layer chooses the element with the highest probability among the values obtained from *Softmax* regression [48] as the classification result. There are classes for open eyes and closed eyes in this research, and when these are labeled as *C*1 and *C*2, the probabilities for the neurons to belong to each of the classes can be expressed as *P*1 and *P*2, respectively. If the *P*1 is larger than the *P*2, *C*1 is selected as final class, and vice versa.

### 2.3. CNN Training

The original neural network was created to imitate the neurons in human brains. The strength of the electrical signals when neurons are connected is expressed as a weight, and when a specific value is inputted to the neuron, it is multiplied by the weight associated with the neuron and the resulting value is used to compute the weighted sum of all of the weights connected to the next layer and used as input for an activation function. In such a network, the actions performed between neurons affect the output neuron when a new value is entered. Back-propagation is a method used to learn the weights in this type of neural network. It has been used for optimization in many machine-learning algorithms.

We use a stochastic gradient descent (SGD) method, one of many back-propagation methods, to “teach” our CNN [49]. The SGD method optimizes weights by computing derivatives after reflecting the differences between desired and calculated outputs. During the training process, an input sample is labeled with the correct class in advance. If the labeled sample passes through the feed-forward stage in the neural network, the desired and calculated outputs obtained from the neural network can either be the same or different. If there is a difference between desired and actual outputs, the learning rate is multiplied with an equation reflecting this difference and the result is applied when the values for the new weights are updated. The filter coefficient in the convolutional layers and the weight values in the fully connected layer of the CNN used in the proposed method are “learned” using this SGD method. Different from the gradient descent (GD) method, the SGD method divides the training set into mini-batches, performs an iteration for each mini-batch, and then proceeds to learn by defining the time it takes for all iterations to complete as 1 epoch. The parameters used for the SGD method in this study are as follows: *mini-batch size* = 10, *learning rate* = 0.001, *learning rate drop factor* = 0.1, *learning rate drop period* = 10, *L2 regularization* = 0.0001, *momentum* = 0.9. The definition of each parameter can be found in [50]. During training, data samples are shuffled and the learning rate is multiplied by *learning rate drop factor* for each 10-epoch period. The weights used in the *FC layer* are initialized randomly using a Gaussian distribution with a mean of 0 and a standard deviation of 0.001. The biases are initialized to 0. We experimentally determined the optimal parameters for SGD method so as to obtain the lowest loss value and the highest accuracy of training data of Figure 6.

The ResNet-50 model trained by our experimental databases has been made public in [51] to allow other researchers to compare and evaluate its performance.

## 3. Experimental Results

### 3.1. Experimental Environment and Data Augmentation

In order to verify the proposed CNN-based open/closed eye classification approach under different DB circumstances, we used combination of database 1 (DB1), built in our lab, and an open database 2 (DB2) in our experiment. 

**Figure 3:**
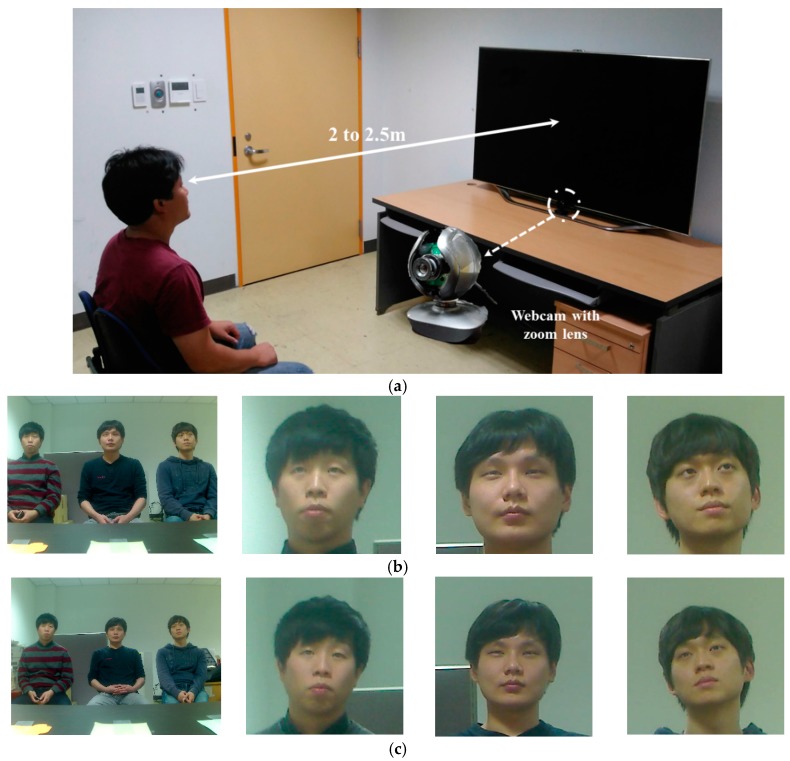
DB1 acquisition environment and obtained images: (**a**) experimental environment; (**b**) obtained images (**left**: Image captured at a distance of 2 m; **right**: The face images cropped from the images on the left); (**c**) obtained images (**left**: the image captured at a distance of 2.5 m, **right**: the face images cropped from the images on the left).

DB1 was created in an environment with a person watching TV indoors, with images taken at distances between 2 and 2.5 m. The camera used for capturing the images was a Logitech C600 web camera [52] with a zoom lens attached. The environment used for image acquisition is shown in Figure 3. We used eye images obtained by extracting facial and eye features from images captured at a resolution of 1600 × 1200 pixels. DB1 has been made public through [51] to allow other researchers to compare and evaluate its performance.

**Figure 4:**
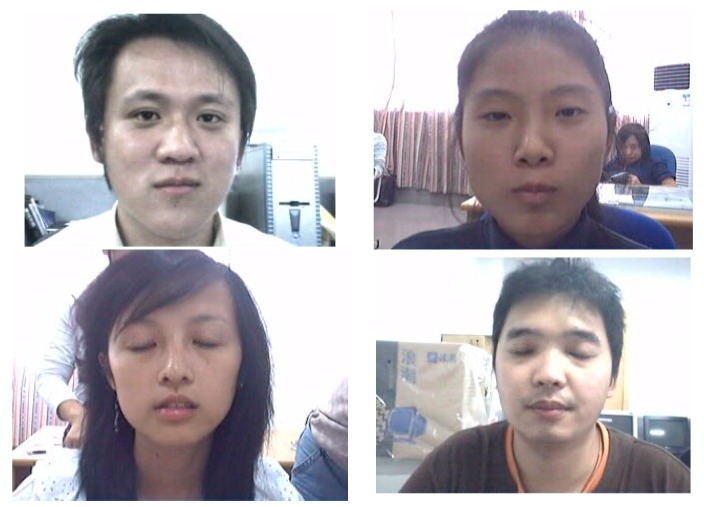
Sample images from DB2.

The eye images in DB2 are composed of images obtained by extracting eye features from 80 video clips from an open database (ZJU Eyeblink Database) [53]. The resolution of the images is 320 × 240 pixels. Examples of the images in DB2 are presented in Figure 4. In our experiments, we used the combined database of DB1 and DB2 for CNN training and testing in order to check the robustness of proposed CNN-based method irrespective of the kinds of databases.

Table 3 shows the descriptions of DB1 and DB2. The reason that the number of closed eye images in DB2 is less than the number of open eye images is because of the small number of closed eye images in the open database (ZJU Eyeblink Database) itself.

**Figure 5:**
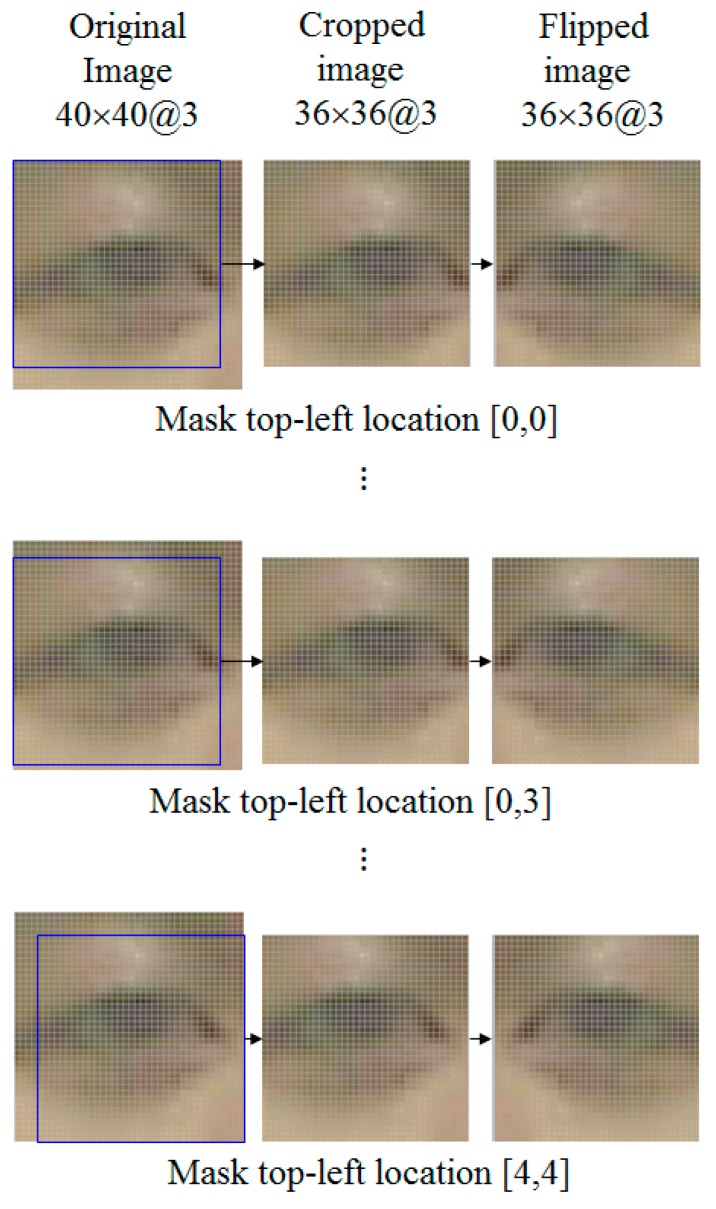
Creating images for augmented DB.

Generally, a substantial amount of data is required to determine optimal values for the numerous coefficients and weights within a CNN structure, and data augmentation techniques are used frequently [34]. This is because it is easy to obtain an overfitting result for the training set if we conduct training and testing with a small amount of data. Therefore, we create an augmented DB as shown in Figure 5 and Table 3. Based on a system of data augmentation using image translation and cropping [34], we created an augmented DB with 610,050 images, by using the original eye images at 40 × 40 pixel resolution (RGB three channels).

We performed data augmentation by cropping the image to a size of 36 × 36 pixels. As shown in Figure 5, we obtained 36 × 36 pixel images while changing the location of the cropping mask. In a 40 × 40 pixel image, the number of applications for the cropping mask can be calculated as follows. We set the cropping mask location to the top-left as a standard, as shown in the first image in Figure 5. All cases where the *x*-coordinate (horizontal location) falls between 0 and 4 are then combined with the cases where the *y*-coordinate falls between 0 and 4, image cropping is applied 25 (5 × 5) times and all 25 cropped images are flipped horizontally so that we can obtain 50 total images from one original image. As a result, we created an augmented DB with 610,050 (12,201 images × 50) images in total as shown in Table 3.

In general, the period of closing eyes is much smaller than that of opening eyes because we close our eyes when blinking. Therefore, the number of closed eye images is much smaller than that of open eye images, which can cause the overfitting of CNN training and unfair evaluation of types 1 and 2 errors. Type 1 errors are those where closed eye images are incorrectly classified as open eye, and type 2 errors are those where open eye images are incorrectly classified as closed eye. Therefore, we gave instructions to participants to intentionally (but naturally) close eyes by longer time than natural blinking when we collected DB1 of Table 3, and the consequent number of closed eye images is larger than that of open eye images in DB1 of Table 3. Nevertheless, the data were not collected when participants were sleeping. From that, we could reduce the difference between the numbers of open and closed eye images in the combined DB (DB1 and DB2 of Table 3), which could lessen the overfitting of CNN training and enable the fair evaluation.

For the experiments in this study, a system with the following specifications was used: A 3.33 GHz Intel^®^ (Santa Clara, CA, USA) Core™ i7-975 CPU, 16 GB RAM, and an NVIDIA (Santa Clara, CA, USA) GeForce GTX 1070 graphics card [54] with a compute unified device architecture (CUDA) 8.0 library [55] that supports parallel processing. The programs were executed on a 64-bit Windows 10 operating system. All the training and testing algorithms for the CNN were implemented using Windows Caffe (version 1) [56].

### 3.2. Training of the Proposed Deep Residual CNN

**Figure 6:**
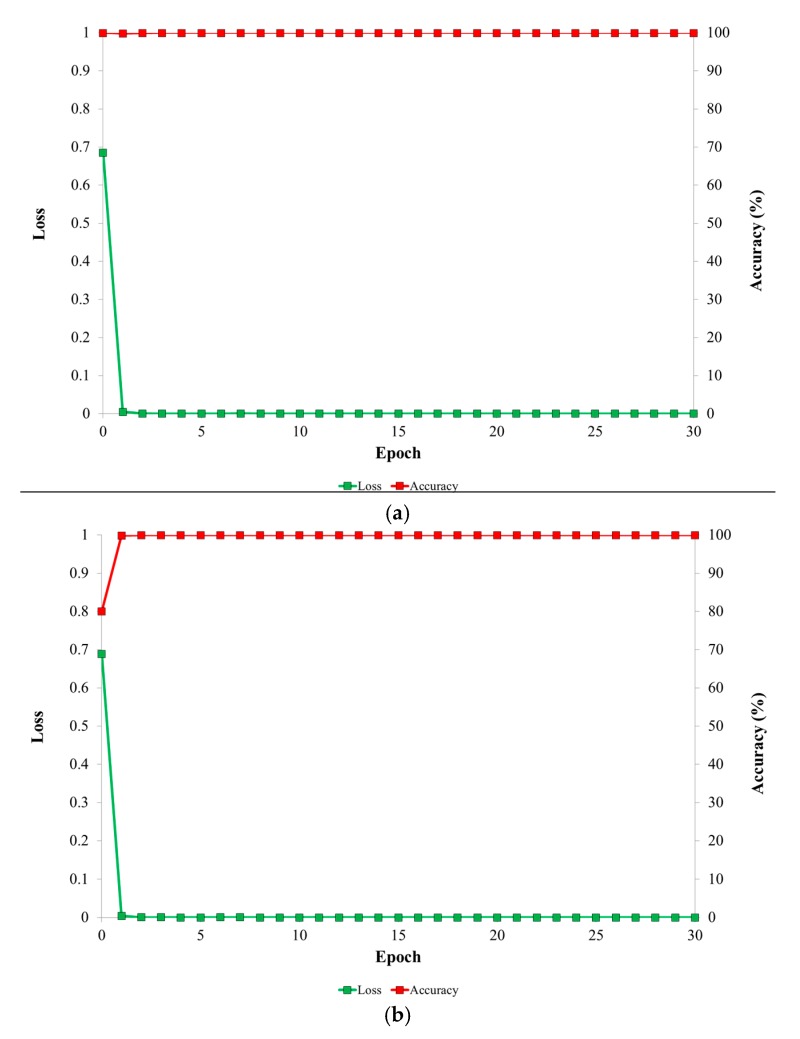
Graphs of loss and accuracy for each epoch during the training procedure. (**a**) The 1st fold of cross validation; (**b**) The 2nd fold of cross validation.

Figure 6 presents graphs of loss and accuracy for each epoch during the training procedure for the 1st and 2nd folds of cross validations. In all cases, the loss values approach 0 while the training accuracy approaches 100% as the number of training epochs increases. This demonstrates that the training of the proposed deep residual CNN is sufficient with our training data. Compared to the conventional training of CNN structure, fine-tuning is based on the pre-trained model, and the convergence speed of loss and accuracy of Figure 6 is faster than that by conventional training.

### 3.3. Testing Accuracies of the Proposed CNN Structure Based on Number of Training Epochs

In order to implement the evaluation of the CNN-based classification system for open and closed eye images introduced in this paper, we performed two-fold cross validation. For training, we used the augmented database of Table 3 whereas the original database of Table 3 was used for testing as shown in Table 4. The final value for accuracy was obtained from the average of the two values of accuracy from two-fold cross validations.

**Figure 7:**
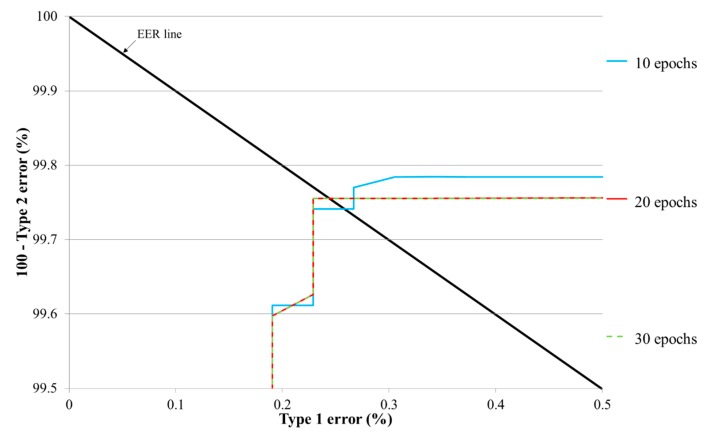
ROC curves for the testing data using the proposed CNN based on the number of training epochs.

Table 5 presents the testing error of the proposed deep residual CNN based on the number of training epochs. The change in number of epochs also changes the learning rate of the SGD method and is related to the filter coefficients in the convolutional layers and the number of weight updates in the fully connected layer. Type 1 and type 2 errors generally have a trade-off relationship; based on the threshold for the CNN output, when type 1 errors increase, type 2 errors decrease, and vice versa (refer to Figure 7). The equal error rate (EER) indicates the average value of type 1 and type 2 errors when the difference between these two errors reaches its minimum. Figure 7 presents the receiver operating characteristic (ROC) curves for type 1 and type 2 errors for the testing data with the proposed method based on the number of training epochs. Table 5 shows the average errors from two-fold cross validation, and Figure 7 represents the average graphs from two-fold cross validation.

Following the experiment, as shown in Table 5 and Figure 7, we found that the case when the number of epochs was 20 or 30 yielded the best performance. Because the smaller number of epoch is preferred in case of same accuracy, we used the CNN model trained based on the number of epoch of 20.

### 3.4. Comparative Experimental Results and Analyses with the Proposed Method and Others

For the next experiment, we compared the testing accuracies for classifying open and closed eye images with the proposed deep residual CNN to other methods. For comparison, we used HOG-SVM [31], fuzzy system-based method [17], the conventional CNN models of AlexNet [34], VGG face [36], and GoogLeNet [38]. The same databases were used for our method and the other methods, and the average accuracies were measured using two-fold cross validation. The size of the input images was normalized to 224 × 224 pixels for AlexNet, VGG face, and GoogLeNet based on previous research [34,36,38].

AlexNet is composed of five convolutional layers and three *FC layers* [34]. AlexNet uses 96 filters of 11 × 11 × 3 in the 1st convolutional layer, and uses a local response normalization (LRN) layer after a ReLU layer. The weights in each layer were initialized to random values based on a Gaussian distribution with a mean of zero and standard deviations of $10^{-2}$ and $10^{-3}$. VGG face is comprised of 13 convolutional layers and 3 *FC layers* [36], which is deeper than AlexNet. One characteristic of VGG face is to include two or three convolutional layers before the pooling layer. In all the convolutional layers, a filter of size 3 × 3 pixels is used. Although the size of the filter used in VGG face is smaller than that of AlexNet, efficient feature extraction is accomplished by VGG face by using two or three convolutional layers before the pooling layer. VGG face was fine-tuned using our experimental databases. GoogLeNet is deeper than VGG face [38], and adopts convolutional filters of various sizes. The main characteristic of GoogLeNet is its use of inception layers. In an inception layer, three convolution computations of size 1 × 1, 3 × 3, 5 × 5, and one max pooling of size 3 × 3 are performed. The weights of each layer were initialized based on the results in [46]. For the training of AlexNet, VGG face, and GoogLeNet, the mini-batch size was set to 25, and the testing accuracies were measured for 10, 20, and 30 training epochs. For non-CNN-based methods, we used HOG-SVM method [31] and fuzzy system-based method [17] for comparison. In case of using HOG-SVM method, from the input images, the features were extracted by HOG, and the classification of open and closed eye images was performed based on SVM using a radial basis function (RBF). The fuzzy system-based method [17] uses the standard deviation of vertical pixel length calculated from binarized image for classification. The binarized image is obtained by fuzzy-system based segmentation. 

**Figure 8:**
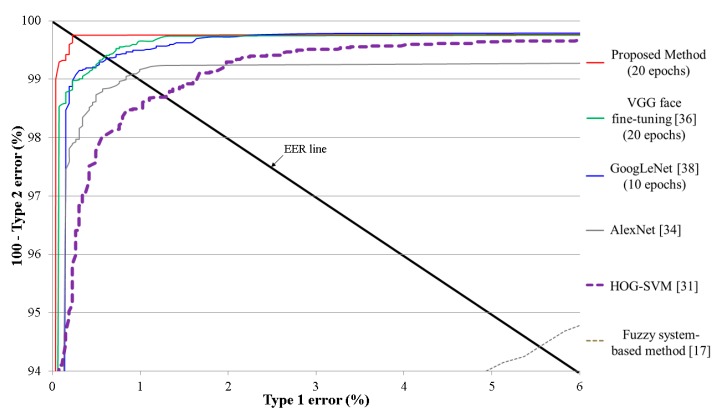
ROC curves for the testing data.

Table 6 shows the average errors from two-fold cross validation, and Figure 8 represents the average graphs from two-fold cross validation. As shown in Table 6 and Figure 8, we find that our proposed method achieves superior testing accuracies compared to HOG-SVM, fuzzy system-based method, AlexNet, VGG face fine-tuning, and GoogLeNet. By using a residual CNN, which is deeper than other CNN models, various factors in the input images could be sufficiently trained using our CNN model, which enables higher classification accuracy for open and closed eye images. In addition, as explained in Section 2.2, because the ResNet-50 CNN model has the characteristics of considering the residual information that is not abstracted by convolutional layers through shortcut of Figure 2, the ResNet-50 CNN model can obtain more accurate filters and classifiers. In addition, this model uses the concept of bottleneck for the convolutional operation, which enables the computational efficiency. Based on these characteristics and deeper structure used in this model, the ResNet-50 CNN model shows higher accuracy of image classification compared to other famous CNN models such as VGG-16 and GoogLeNet, etc. [39], which was also confirmed by our comparative experiments of Table 6 and Table 7 and Figure 8.

AlexNet shows the higher accuracy than HOG-SVM because more optimal filters could be obtained by CNN training than HOG-SVM which uses the manually determined HOG filters. Nevertheless, the accuracy by AlexNet is lower than GoogLeNet and VGG face fine-tuning because the GoogLeNet and VGG have deeper structure than AlexNet. In general, if training is successfully done, the deeper CNN structure can produce more accurate features and better classification accuracy. Therefore, the GoogLeNet and VGG face fine-tuning outperform the AlexNet.

In Table 7, we present summarized comparative results for our method and the other methods. One can see that our method outperforms the other methods.

**Figure 9:**
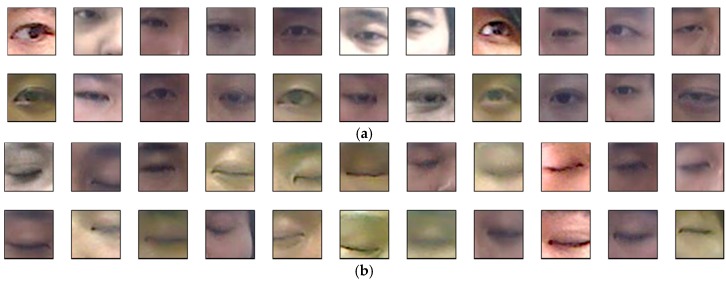
Examples of correct classification of open and closed eye images using the proposed method. (**a**) Open eye images; (**b**) Close eye images.

In Figure 9, we provide examples of correct classification of open and closed eye images when using our method. As shown in this figure, although the images have various illumination and different eye size, our method can classify open and closed eye images correctly.

**Figure 10:**
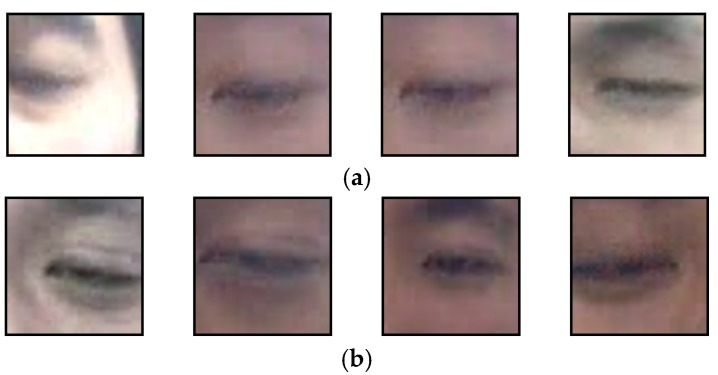
Error images from the classification of open and closed eye images using the proposed method. (**a**) Type 1 error; (**b**) Type 2 error.

Figure 10a contains type 1 error images from the classification of open and closed eyes using the proposed CNN method. The type 1 error cases are caused by image blurring, shadow, and the inclusion of eyebrows. Figure 10b contains type 2 error images. The type 2 error cases are caused by image blurring, illumination variation, and the inclusion of eyeshadows. In case that user opens his or her eye slightly, the type 2 error case occurs, also.

For the next experiment, we measured the average processing time per image for our method. As shown in Table 8, we found that our system can operate at a speed of 28.2 frames per second (1000/35.41). When using our method for video-based classification, multiple images can be processed in parallel, greatly enhancing the processing speed.

**Figure 11:**
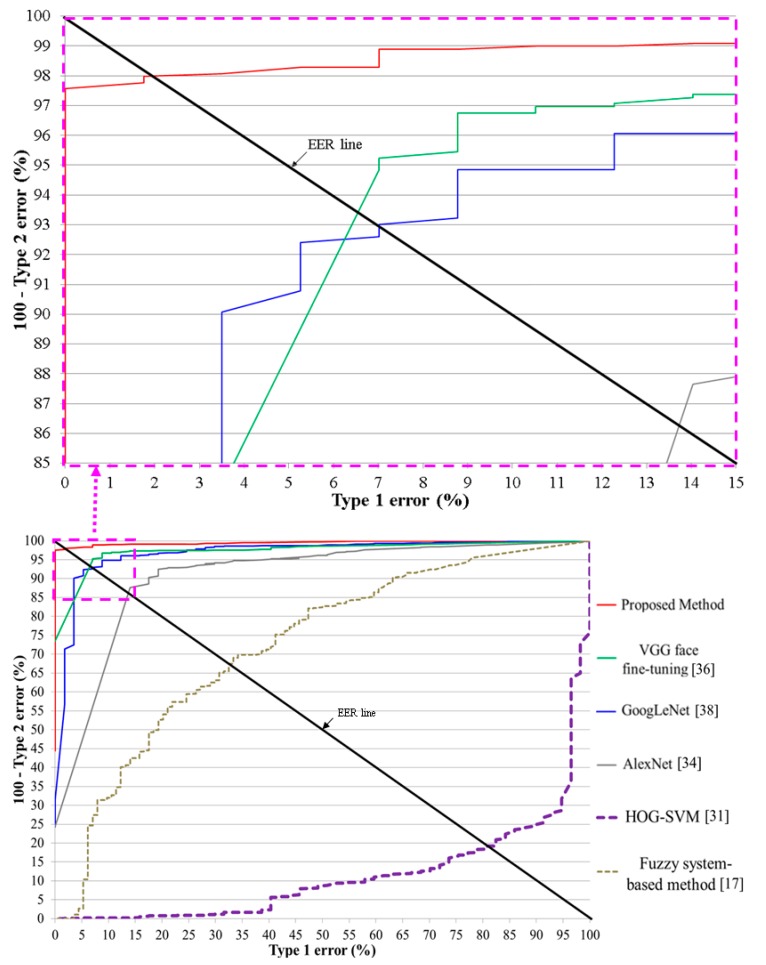
ROC curves with open database of near-infrared (NIR) eye images.

We performed the additional experiments with the open database of NIR eye images [18] for the evaluation of the proposed algorithm. As shown in Table 9 and Figure 11, our method outperforms other methods with this open database, and the EER of classifying open and closed eyes is about 1.89% by our method.

**Figure 12:**
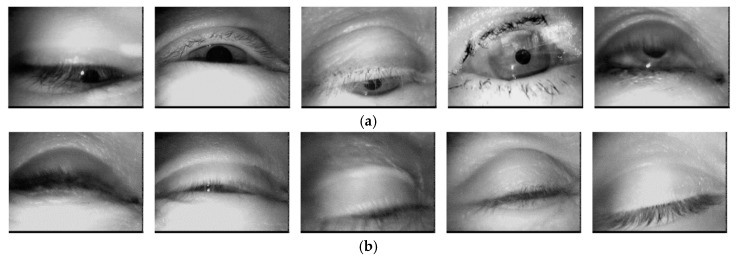
Examples of correct classification of open and closed eye images of open database of NIR eye images using the proposed method. (**a**) Open eye images; (**b**) Closed eye images.

Figure 12 shows the examples of correct classification of open and closed eye images of open database of NIR eye images [18] when using our method. Even in the case of narrow open eye, open eye including the reflection of eye glasses surface and motion blurring by eye closing, our method can correctly classify them as shown in Figure 12a. As shown in Figure 12b, even in the case of shadow by double eyelid or long eyelashes, they can be correctly classified.

**Figure 13:**
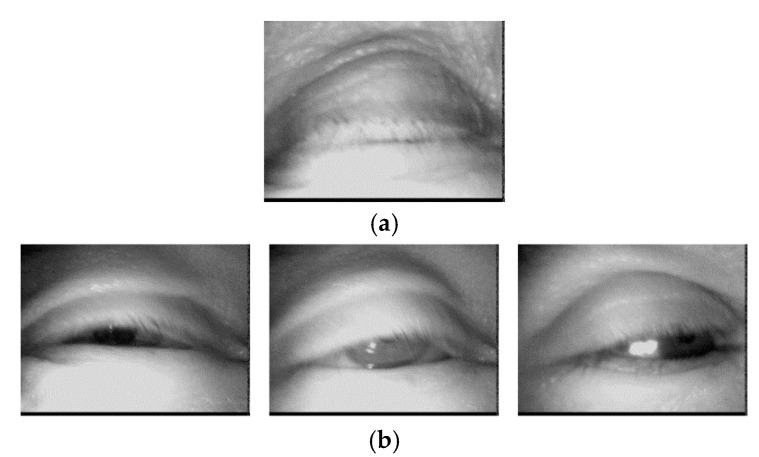
Error images from the classification of open and closed eye images of open database of NIR eye images using the proposed method. (**a**) Type 1 error; (**b**) type 2 error.

Figure 13 shows the examples of classification errors of open and closed eye images of open database of NIR eye images [18] when using our method. As shown in Figure 13a, type 1 error is caused by little eyelash and shadow by skin wrinkle in the image. Type 2 error is caused by severely narrow open eye where pupil is almost invisible by eyelid as shown in Figure 13b.

## 4. Conclusions

We have proposed a method for the classification of open and closed eye images using a deep residual CNN structure. For CNN training and testing, a combined DB was used, with images collected from two different environments. The proposed method achieved a lower EER for the classification of open and closed eye images compared to other CNN structures (AlexNet, VGG face, and GoogLeNet) and other non-CNN based methods. The type 1 and 2 error cases are caused by image blurring, shadow, illumination variation, the inclusion of eyebrows, and narrow eye. Our collected DB1 has been made public through [51] to allow other researchers to compare and evaluate its performance. Although the CNN-based classification shows high accuracy in various fields, the main disadvantage of the CNN-based method is that intensive training is required, which takes much processing time with lots of training data. However, in actual experimental environment, it is often the case to have difficulty in collecting lots of training data, and data augmentation is performed, consequently. Therefore, in order to reduce the time-consuming procedure of CNN training, various CNN models trained in our research are made public through [51] to allow other researchers to compare and evaluate its performance, also.

In the future, we plan to improve classification accuracy by developing a video-based method as an extension of the single image-based method. Additionally, we wish to verify the validity of the proposed method for eye images obtained from near-infrared or thermal cameras. 

## Figures and Tables

**Table 1:** Comparison of existing methods and the proposed method.

Category		Method	Advantages	Drawbacks
Non-image-based		EOG [6,7], EMG [8,9]	Fast data acquisition speed.	- Inconvenient because sensors have to be attached to a user. - Low accuracy if noise from the movements of a user are included. Thus, there are limitations on user behavior.
Image-based	Video-based	Uses optical flow, SIFT, difference images [11], and eyelid and facial movements [12]. Calculates the difference in the number of black pixels in the eye areas in consecutive frames [13].	High accuracy because it uses information from several images in a videos.	Long processing time due to working with several images.
	Single image-based (non-training-based)	Iris detection-based [14], sub-block-based template matching and ellipse-fitting-based method [15], template-matching-based method [16], fuzzy-system-based method [17], approximation of the lower and upper eyelids based on a likelihood map [18], and oriented edges [19].	Classifying open/closed eyes from a single image without any additional training process.	Low classification accuracy if vague features are extracted because the information about open/closed eyes is extracted from a single image. Therefore, there are restrictions on the environment in terms of obtaining optimal images for feature extraction.
	Single image-based (training-based)	SVM-based [22], HOG-SVM based [31], AAM-based [23], user dependent eye template-based matching [25,26], tensor PCA-based [27], neural network and probability density functions-based [28], PCA-based [29], and trained Adaboost with CC-based [32].	- Shorter processing time than that of video-based methods. - Relatively high accuracy in classifying open/closed eyes compared to single-image-based (non-training-based) methods.	- Difficult to extract accurate features with the AAM method in cases with complex backgrounds or far away faces, leading to low resolution in the image. - Difficult to find an optimal feature extraction method and feature dimension for extracting the features used as the input for a classifier.
		CNN-based **(proposed method)**	- The filters and classifiers for optimal feature extraction can be automatically obtained from the training data without pre- or post-processing. - Condition-change-enduring classification performance due to a large-capacity DB learning under different conditions (image capture distance, image resolution, image blurring, and lighting changes).	- Requires a learning process from a large-capacity DB.

**Table 2:** Output size, numbers and sizes of filters, number of strides, and padding in our deep residual CNN structure (3* represents that 3 pixels are included as padding in left, right, up, and down positions of input image of 224 × 224 × 3 whereas 1* shows that 1 pixel is included as padding in left, right, up, and down positions of feature map) (2/1** means 2 at the 1st iteration and 1 from the 2nd iteration).

Layer Name		Size of Feature Map	Number of Filters	Size of Filters	Number of Strides	Amount of Padding	Number of Iterations
*Image input layer*		224 (height) × 224 (width) × 3 (channel)					
*Conv1*		112 × 112 × 64	64	7 × 7 × 3	2	3*	1
*Max pool*		56 × 56 × 64	1	3 × 3	2	0	1
*Conv2*	*Conv2-1*	56 × 56 × 64	64	1 × 1 × 64	1	0	3
	*Conv2-2*	56 × 56 × 64	64	3 × 3 × 64	1	1*	
	*Conv2-3*	56 × 56 × 256	256	1 × 1 × 64	1	0	
	*Conv2-4 (Shortcut)*	56 × 56 × 256	256	1 × 1 × 64	1	0	
*Conv3*	*Conv3-1*	28 × 28 × 128	128	1 × 1 × 256	2/1**	0	4
	*Conv3-2 (Bottleneck)*	28 × 28 × 128	128	3 × 3 × 128	1	1*	
	*Conv3-3*	28 × 28 × 512	512	1 × 1 × 128	1	0	
	*Conv3-4 (Shortcut)*	28 × 28 × 512	512	1 × 1 × 256	2	0	
*Conv4*	*Conv4-1*	14 × 14 × 256	256	1 × 1 × 512	2/1**	0	6
	*Conv4-2 (Bottleneck)*	14 × 14 × 256	256	3 × 3 × 256	1	1*	
	*Conv4-3*	14 × 14 × 1024	1024	1 × 1 × 256	1	0	
	*Conv4-4 (Shortcut)*	14 × 14 × 1024	1024	1 × 1 × 512	2	0	
*Conv5*	*Conv5-1*	7 × 7 × 512	512	1 × 1 × 1024	2/1**	0	3
	*Conv5-2 (Bottleneck)*	7 × 7 × 512	512	3 × 3 × 512	1	1*	
	*Conv5-3*	7 × 7 × 2048	2048	1 × 1 × 512	1	0	
	*Conv5-4 (Shortcut)*	7 × 7 × 2048	2048	1 × 1 × 1024	2	0	
*AVG pool*		1 × 1 × 2048	1	7 × 7	1	0	1
*FC layer*		2					1
*Softmax*		2					1

**Table 3:** Descriptions of original and augmented databases.

Kinds of Image	Original Database			Augmented Database		
	DB1	DB2	Combined DB	Augmented DB1	Augmented DB2	Combined Augmented DB
Open eye images	2062	4891	6953	103,100	244,550	347,650
Closed eye images	4763	485	5248	238,150	24,250	262,400
Total	6825	5376	12,201	341,250	268,800	610,050

**Table 4:** Descriptions of training and testing databases in two-fold cross validation.

Kinds of Image	1st-Fold Cross Validation		2nd-Fold Cross Validation	
	Training (Augmented Database)	Testing (Original Database)	Training (Augmented Database)	Testing (Original Database)
Open eye images	173,850	3476	173,800	3477
Closed eye images	131,150	2625	131,250	2623
Total	305,000	6101	305,050	6100

**Table 5:** Testing results from the proposed CNN classification system for open/closed eyes based on a changing number of training epochs (unit: %).

# of Epochs	Type 1 Error	Type 2 Error	EER
10	0.26687	0.25888	0.26288
20	**0.22875**	**0.2445**	**0.23663**
30	0.22875	0.2445	0.23663

**Table 6:** Comparative testing errors for our method and other methods (unit: %).

Method	# of Epochs	Type 1 Error	Type 2 Error	EER
Fuzzy system-based method [17]	N/A	5.55	5.58	5.565
HOG-SVM [31]	N/A	1.29623	1.29416	1.2952
AlexNet [34]	10	**0.91498**	**0.90617**	**0.91058**
	20	0.91498	0.93494	0.92496
	30	0.91498	0.93494	0.92496
GoogLeNet [38]	10	**0.64811**	**0.64718**	**0.64765**
	20	0.68624	0.66157	0.67391
	30	0.68624	0.66157	0.67391
VGG face fine-tuning [36]	10	0.60999	0.61832	0.61416
	20	**0.60999**	**0.60405**	**0.60702**
	30	0.60999	0.60405	0.60702
Proposed method	10	0.26687	0.25888	0.26288
	20	**0.22875**	**0.2445**	**0.23663**
	30	0.22875	0.2445	0.23663

**Table 7:** Summarized comparative errors for our method and the other methods (unit: %).

Methods	EER
Fuzzy system-based method [17]	5.565
HOG-SVM [31]	1.2952
AlexNet [34]	0.91058
GoogLeNet [38]	0.64765
VGG face fine-tuning [36]	0.60702
Proposed method	0.23663

**Table 8:** Average processing time per image for the classification of open and closed eye images using the proposed method (unit: ms).

Method	Average Processing Time
Proposed method	35.41

**Table 9:** Comparative errors for our method and the other methods with open database of NIR eye images [18] (unit: %).

Methods	EER
Fuzzy system-based method [17]	32.525
HOG-SVM [31]	80.73549
AlexNet [34]	13.19163
GoogLeNet [38]	7.00067
VGG face fine-tuning [36]	6.08974
Proposed method	1.88934
